# Case Report: Thromboelastography for uremic thrombocytopathy in a patient with COVID-19

**DOI:** 10.3389/fneph.2022.926313

**Published:** 2022-08-19

**Authors:** Lakshmi Kannan, Rishi Raj

**Affiliations:** Department of Internal Medicine, Division of Nephrology, Pikeville Medical Center, Pikeville, KY, United States

**Keywords:** uremia, thrombocytopathy, dialysis, thromboelastography, bleeding

## Abstract

Uremia causes several biochemical and physiological impairments that result in the accumulation of toxins with multiple clinical effects. Bleeding is one of the most common complications of acute and chronic renal failure. The pathogenesis of uremic bleeding is multifactorial, of which uremic thrombocytopathy is the most described clinically. Various tests have been used to evaluate bleeding diathesis in these patients including bleeding time, prothrombin time, activated partial thromboplastin time, and international normalized ratio, but there are only a few studies that use thromboelastography as a point-of-care test to identify platelet dysfunction. In addition, COVID-19 increases hemorrhagic complications due to platelet dysfunction or hemostasis exhaustion. COVID-19 could also potentially cause platelet dysfunction as a secondary consequence of acute kidney injury. There are only a few studies reporting the use of thromboelastography in COVID-19–induced hypercoagulability, but not in diagnosing or managing platelet-related abnormalities. We present a patient with COVID-19 who developed acute kidney injury in the hospital and retroperitoneal hemorrhage from uremic platelet dysfunction. We used point-of-care thromboelastography with platelet mapping to determine uremic platelet dysfunction.

## Introduction

Patients with acute and chronic renal failure are predisposed to bleeding as a result of platelet dysfunction, anemia, drug accumulation due to poor clearance, and dialysis itself in patients requiring it (1). Commonly, mucosal, serosal, or cutaneous bleeding is seen, but intracranial and gastrointestinal bleeding is also reported (2). The exact pathophysiology of uremic bleeding is unknown, but platelet dysfunction–impaired platelet aggregation and adhesion have been implicated (3, 4).

Among patients infected with severe acute respiratory syndrome coronavirus 2 (SARS-CoV-2), coronavirus disease 2019 (COVID-19)-induced coagulopathy has been the most frequently reported. Hemorrhagic complications remain underreported in hospitalized patients with COVID-19 at about 8% (5). One of the major causes is that patients with COVID-19 demonstrate some degree of hypercoagulability necessitating the use of intermediate or therapeutic doses of lower molecular heparin (LMWH) or unfractionated heparin (UFH) (6).

We report a patient who was initially hospitalized with COVID-19 infection who developed acute kidney injury. His hospital course was complicated by retroperitoneal bleeding thought to be secondary to uremic dysfunction/from heparin thromboprophylaxis and COVID-19–induced hemostatic abnormality. We used thromboelastography (TEG) with platelet mapping to provide further insight into the cause of bleeding, which showed uremic thrombocytopathy that was treated with hemodialysis.

This case report shows that point-of-care thromboelastography can be used in patients with acute kidney injury and COVID-19, as monitoring hemostatic competence can be quite challenging.

## Case presentation

**Figure 1:**
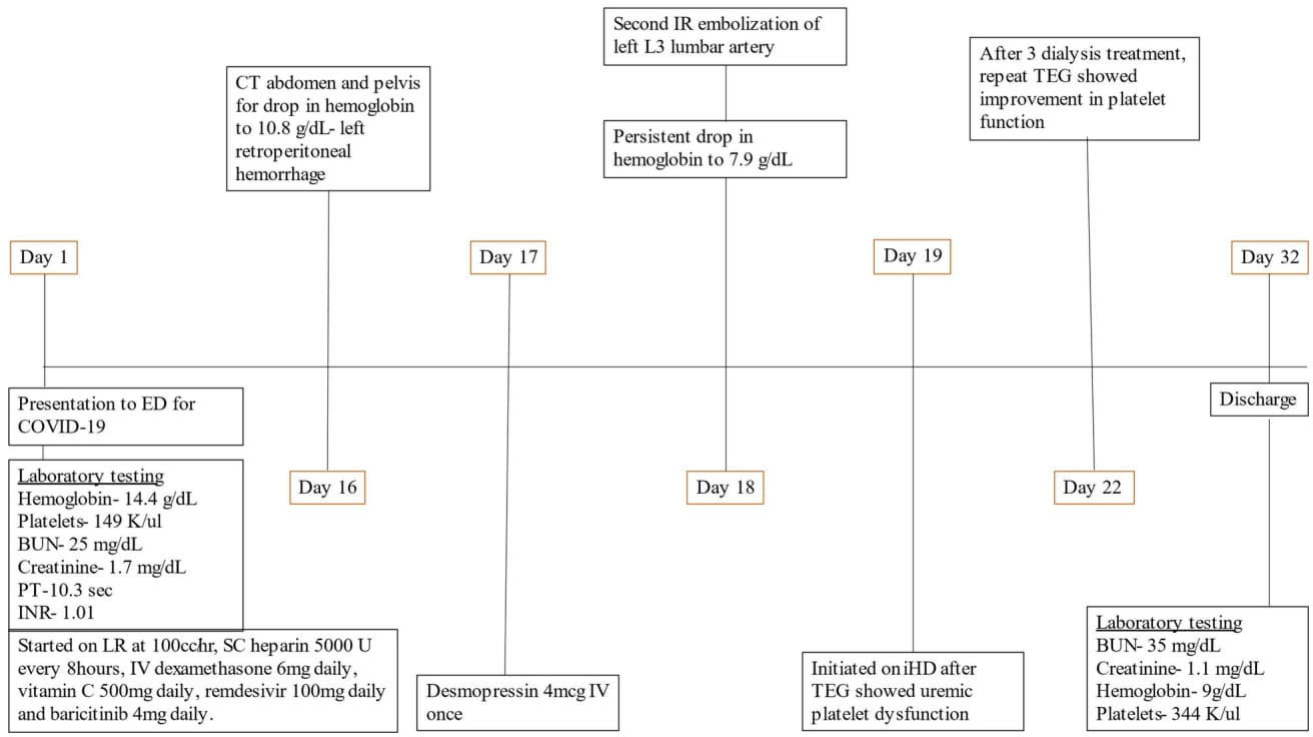
Timeline of patient events. BUN, blood urea nitrogen; ED, emergency department; PT, prothrombin time; COVID-19, coronavirus disease 19; INR, international normalized ratio; LR, ringer lactate; SC, subcutaneous; CT, computed tomography; IR, interventional radiology; iHD, intermittent hemodialysis; TEG, thromboelastography.

A 56-year-old white man presented to the emergency department (ED) for an evaluation of cough, dyspnea during exertion, and sore throat that began 3 days before presentation (**Figure 1**). The patient had a personal medical history of hypothyroidism, hyperlipidemia, and asthma. There is no medical history of renal disease, and the patient is not on antiplatelets or anticoagulants. His surgical history is only significant for cholecystectomy. Laboratory testing conducted in the ED came back with a positive result for COVID-19 and elevated serum blood urea nitrogen (BUN) of 25 mg/dl and creatinine of 1.7 mg/dl. Physical examination was notable for hypoxia, saturating at 86% with 4 L of nasal cannula oxygen. The patient was placed on bilevel positive airway pressure (BiPAP) and admitted to the intensive care unit (ICU) for closer monitoring and management.

The patient was started on intravenous (IV) fluids with lactated Ringer’s at 100 ml/h for elevated creatinine. Despite being on BiPAP, the patient had worsening hypoxic respiratory failure and was intubated and placed on mechanical ventilation. He was also started on a COVID-19 treatment pathway with IV dexamethasone 6 mg daily, vitamin C 500 mg daily, remdesivir 100 mg daily, and baricitinib 4 mg daily.

**Figure 2:**
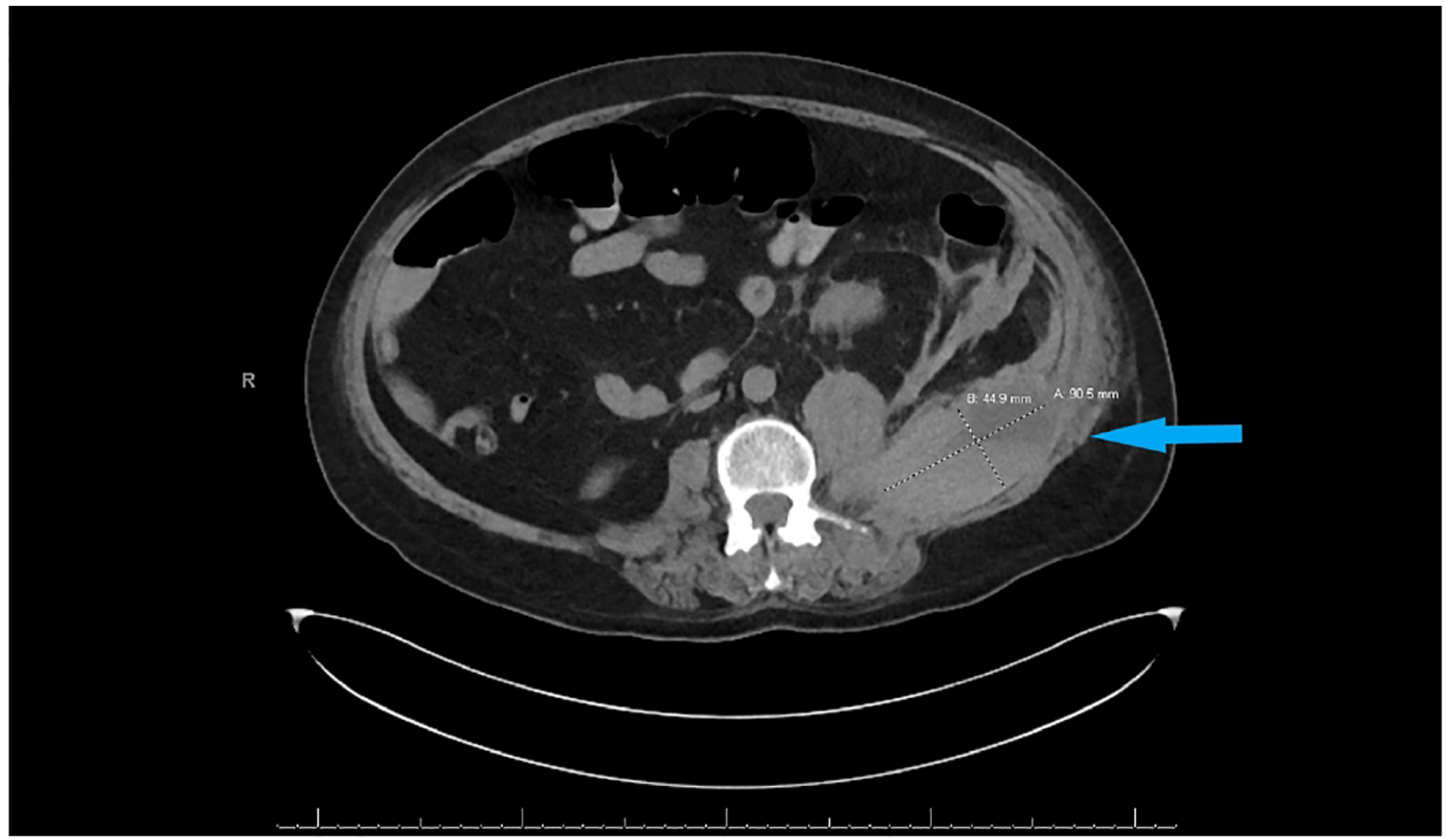
CT abdomen and pelvis without contrast showing left retroperitoneal hemorrhage behind the left psoas muscle (blue arrow). Subcutaneous heparin was discontinued, and the patient received 2 units of packed red blood cell transfusion (PRBC) and underwent angiogram with gel foam embolization of the left L3 lumbar artery. Since the patient continued to have a persistent drop in hemoglobin to 7.9 g/dl 2 days after embolization, he was taken back to the operating room for a second embolization. Between the first and second embolization, he received desmopressin 4 mcg IV once as his BUN was 118 mg/dl. Although he underwent a second embolization, point-of-care thromboelastography (TEG) with platelet mapping in the presence of heparinase was performed as the suspicion of uremic platelet dysfunction was high. It showed decreased platelet aggregation (11.9) and increased inhibition (68.1), as shown in **Figure 3A** confirming our diagnosis. Hemodialysis was initiated, and after three dialysis treatments, TEG was repeated, showing an improvement in the parameters (**Figure 3B**).

Subcutaneous heparin 5,000 units every 8 h was administered from hospital day 1 for thromboprophylaxis. The patient was extubated on day 10 of hospitalization and remained stable until day 16 when he became hypotensive with blood pressure of 88/45 mm Hg, hemoglobin had dropped to 10.8 g/day, and creatinine went up to 3.3 mg/dl as shown in **Table 1**. He underwent contrast-free computed tomography (CT) of the abdomen and pelvis that showed left retroperitoneal hemorrhage posterior to the left psoas measuring 9.05 cm × 4.4 cm × 3.3 cm, as shown in **Figure 2**.

**Table 1:** Laboratory results on different days of hospitalization.

	Normal range	Day 1	Day 16	Day 17	Day 18	Day 19	Day 22	Day 32
Hemoglobin and hematocrit (g/dl, %)	11.0-17.8, 32.0- 51.6	14.4, 41.8	10.8, 32.7	9.9, 28.8	7.9, 23.3	9.7, 28.6	8.7, 25.8	9, 26.7
Platelets (K/μl)	134-412	149	317	349	296	249	260	344
BUN (mg/dl)	7-18	25	115	118	128	135	20	35
Creatinine (mg/dl)	0.60-1.30	1.7	3.3	3.4	3.8	4.2	1.1	1.1
PT (s)	9.4- 11.0	10.3	14	11	10.9	10.3	10.5	10.3
INR	0.91- 1.09	1.01	1.5	1.08	1.08	1.01	1.03	1.01

BUN, blood urea nitrogen; PT, prothrombin time; INR, international normalized ratio.

**Figure 3:**
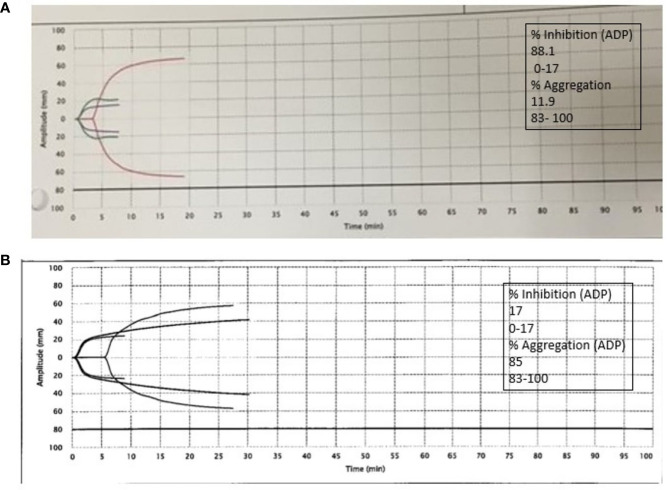
**(A)** Platelet thromboelastography with platelet mapping in the presence of heparinase showing reduced aggregation and increased inhibition. **(B)** After dialysis, platelet mapping shows improvement in percent inhibition and aggregation.

## Discussion

Thromboelastography (TEG) was first developed and described by Dr. Hellmut Hartert at the University of Heidelberg (Germany) in 1948 (7). Its first reported application was to guide blood transfusions in injured soldiers in the Vietnam War. Since then, point-of-care TEG has evolved into a more commonly used test to assess clotting, and it offers an opportunity for an expediated assessment of coagulopathies like thrombocytopenia, factor deficiencies, heparin effect, hypofibrinogenemia, and hyperfibrinolysis (8).

TEG has been shown to be effective in optimizing blood product selection and utilization. It has improved results during surgical procedures such as cardiothoracic surgery and liver transplantation (9). Hence, the National Institute for Health and Care Excellence (NICE) guidelines recommend thromboelastography to help detect, manage, and monitor hemostasis in cardiac surgery patients. Other clinical guidelines do not strongly recommend TEG for use in other settings due to lack of high-quality evidence.

Critically ill patients, in general, are predisposed to bleeding from clinical conditions such as thrombocytopenia, platelet dysfunction, coagulation factor deficiencies, the use of antiplatelet or anticoagulant agents, and invasive procedures. During the global COVID-19 pandemic, multiple cases of venous and arterial thrombotic complications have been reported. Hemorrhagic complications are only rarely reported, raising concern for the use of heparin in hospitalized patients with COVID-19 (10), as the American Society of Hematology recommends standard prophylactic dose anticoagulation for all patients diagnosed with COVID-19 with therapeutic anticoagulation only for patients with documented venous thromboembolism (11).

In addition to coagulopathic complications related to COVID-19 infection itself, it increases the risk of acute kidney injury from relative hypoperfusion of the kidneys from sepsis/shock, alteration of microcirculation from cytokine storm, direct tubular epithelial cell damage, hypoxemia, and systemic acidosis, which influence kidney vascular resistance and direct or indirect toxicity of medications. These patients can accumulate uremic toxins that can lead to platelet dysfunction or uremic thrombocytopathy (12).

In our patient, after an initial period of hypoxia with oxygen saturation at 70%–80% on BiPAP requiring intubation, his oxygen saturation remained relatively stable throughout the hospital course even after extubation, requiring only up to 2–3 L of oxygen.

As shown in multiple studies, platelet dysfunction in uremic patients can occur with abnormalities in the concentration of intracellular adenosine diphosphate, serotonin, and cyclic adenosine monophosphate, the release of platelet α granules, calcium ion mobilization, arachidonic acid metabolism, cyclooxygenase activity, and GPIIb/IIIa binding. Uremic toxins likely responsible are urea, creatinine, methylguanidine, phenol and phenolic acids, and guanidinosuccinic acid (13).

Bleeding in these patients cannot be accurately predicted or assessed based on laboratory evidence of elevated prothrombin time or INR, as it does not adequately characterize multifaceted platelet activity (14).

In this patient, we observed that the patient developed retroperitoneal bleed from three likely possibilities: use of heparin for thromboprophylaxis, hemostatic dysregulation associated with COVID-19, and uremic thrombocytopathy. We performed TEG in the presence of heparinase as this patient was on heparin for thromboprophylaxis, and the results proved uremic thrombocytopathy as the cause of bleeding, eventually leading to hemodialysis treatment and improvement in platelet mapping parameters. We showed that the management of a bleeding complication in a COVID-19 patient with uremia with TEG can improve the clinical diagnosis and potentially help achieve better outcomes.

## Conclusion

This case highlights the fact that the possibility of uremic bleeding associated with kidney injury in COVID-19 infection is higher, which reiterates the use of TEG with platelet mapping as a point-of-care testing modality to accurately delineate patient-specific COVID-19 coagulopathy and platelet-related contribution, keeping in mind the possible limitations including abnormalities in coagulation cascade components other than platelets and patients on anticoagulants and antiplatelet agents.

## Data availability statement

The original contributions presented in the study are included in the article/supplementary material. Further inquiries can be directed to the corresponding author.

## Ethics statement

Written informed consent was obtained from the individual(s) for the publication of any potentially identifiable images or data included in this article.

## Author contributions

LK contributed to the design of the manuscript, case presentation, and discussion. RR contributed to the introduction and review of the manuscript. All authors contributed to the article and approved the submitted version.

## Acknowledgments

The author would like to acknowledge funding support for the publication of this case report, which was provided by the education department at Pikeville Medical Center, KY.

## Conflict of interest

The authors declare that the research was conducted in the absence of any commercial or financial relationships that could be construed as a potential conflict of interest.

## Publisher’s note

All claims expressed in this article are solely those of the authors and do not necessarily represent those of their affiliated organizations, or those of the publisher, the editors and the reviewers. Any product that may be evaluated in this article, or claim that may be made by its manufacturer, is not guaranteed or endorsed by the publisher.
